# Prolene suture-assisted cystoscopic removal of double J ureteral stents in infants

**DOI:** 10.3389/fped.2025.1555402

**Published:** 2025-02-26

**Authors:** Xinhao Zeng, Na Lian, Tong Liu, Xiaoyong Wang

**Affiliations:** ^1^ Department of Pediatric Surgery, Children's Medical Center, The Affiliated Hospital of Southwest Medical University, Luzhou, Sichuan, China; ^2^ Sichuan Clinical Research Center for Birth Defects, Luzhou, Sichuan, China; ^3^ Department of TCM Surgery, The Affiliated Traditional Chinese Medicine Hospital of Southwest Medical University, Luzhou, Sichuan, China

**Keywords:** cystoscopy, double J stent, infant, prolene suture, hydronephrosis

## Abstract

**Objectives:**

The purpose of this study was to describe the prolene suture-assisted cystoscopic removal of double J ureteral stents in infants and summarize the safety, operability, and effectiveness of this approach.

**Methods:**

The clinic data of patients who underwent double J ureteral stent removal by prolene suture-assisted cystoscopy were reviewed. The operation procedure was as follows: First, a folded 4-0 prolene suture was preset into the cystoscope sheath, and the telescope was placed. Second, the cystoscopy was performed by inserting the cystoscope into the urinary bladder and finding the end of the double J ureteral stent under cystoscopy. Subsequently, the folded suture was pushed out of the sheath to form a coil that was used to hook 1–2 cm of the end of the stent. Finally, the stent was caught by tightening the prolene coil while the cystoscope was retracted into the cystoscope sheath; the removal was accompanied by exiting the cystoscope. Cystoscopy was repeated to confirm no additional damage.

**Results:**

Overall, 15 double J ureteral stents were retrieved in 15 infants, whose average age was 3.78 ± 1.2 months, average weight was 5,951 ± 797 g, average residence time of the stents in the ureter was 31.20 ± 2.14 days, and the average operation time was 3.5 ± 1.2 min. No complications, such as urethral injury, occurred during the operation.

**Conclusions:**

Prolene suture-assisted cystoscopy is one of the simple, safe, and effective technique for the removal of double J ureteral stents, especially suitable for infants or patient in whom the grasping forceps cannot pass through the matching cystoscope sheath.

## Introduction

Double J ureteral stent (DJUS), a commonly used ureteral stent, is widely retained in infant patients after pyeloplasty and cystoureteroplasty, and is removed by using grasping forceps under cystoscopy at 4–6 weeks after the said operation. However, in some young infants with a small urethra, especially male infants, the grasping forceps cannot pass through the matching cystoscope sheath, or a suitable cystoscope is not available. Some advanced techniques attempted to remove a DJUS have been reported in the literature (1–3). Nevertheless, these techniques have been associated with adverse effects, such as additional damage, infection, discomfort, and failure of stent removal. Herein, we describe a hitherto unreported technique involving the use of prolene suture for the removal of a DJUS; we believe the technique affords the advantages of simplicity, effectiveness, and safety, and would be a good option for stent removal.

## Methods

This study was approved by the ethics committee of the Affiliated Hospital of Southwest Medical University (KY2023002). Clinical data of infants who underwent surgery for DJUS removal from January 2019 to June 2022 were reviewed. The inclusion criteria: (1) The ureteral stents were confirmed to be in proper position by x-ray and ultrasound; (2) no indicator of infection was detected in the blood and urine routine examination; and (3) the infant's urethra was too small for the use of a cystoscope with an operating channel for grasping forceps. Written informed consent was obtained from all infants' parents.

**Figure 1:**
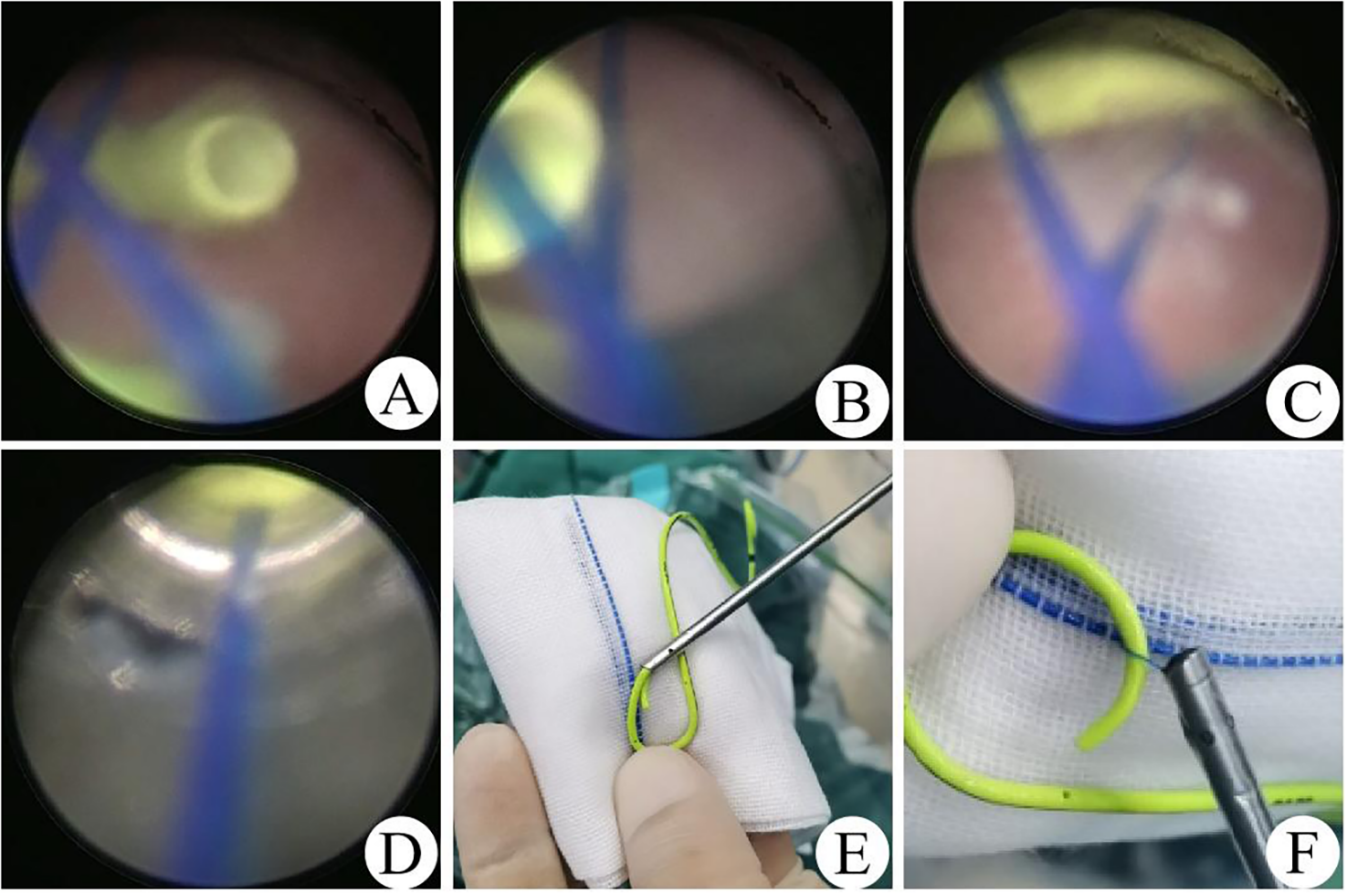
The process of prolene suture-assisted cystoscopic removal DJUS. **(A)** Find the end of the DJUS under cystoscopy. **(B,C)** Hook the end of the DJUS. **(D)** Tighten the prolene coil to the cystoscope sheath. **(E)** DJUS removal. **(F)** Grasp the DJUS.

The patient was placed in a lithotomy position after being administered a combination of intravenous and inhalation anesthesia (0.5–2 mg/kg ketamine) followed by routine disinfection and towel laying. During the procedure, a folded prolene suture was preset into the cystoscope sheath (Hawk 8Fr, N4009), with the coil facing forward, and retrograde cystoscopy (Hawk, T0193) was performed to find the end of the DJUS after injecting tetracaine hydrochloride mortar into the urethra for lubrication. The coil was pushed into the bladder by sliding the telescope and hooked to 1–2 cm of the end of DJUS under cystoscopy. The DJUS was caught by tightening the prolene coil while the cystoscope was retracted into the cystoscope sheath and removed completely accompanied by exiting the cystoscope sheath. A 6Fr catheter was placed into the bladder to drain urine after performing repeated cystoscopy to confirm no additional damage (Figure 1).

All patients were sent to the postanesthesia care unit after the procedure. The operation time was calculated from the folding of the prolene suture until the removal of the DJUS. All patients were discharged and sent home after the removal of the urinary catheter on the second day of the operation.

## Results

A total of 15 patients, i.e., 15 boys, with an average age of 3.78 ± 1.2 months and an average weight of 5,951 ± 797 g were enrolled were enrolled, and they were underwent Laparoscopic Anderson-Hynes operation for the treatment of ureteropelvic junction obstruction (UPJO) due to grade 3 or higher hydronephrosis, meanwhile the DJUSs were implanted to prevent anastomotic stenosis and drain urine. After an average of 31.20 ± 2.14 days, 15 DJUSs were removed by prolene suture-assisted cystoscopy. The mean operation time was 3.5 ± 1.2 min (Table 1). There were no complications such as a urethral laceration or bladder perforation in any case.

**Table 1:** Patients’ basic information and characteristics.

Case number	Gender	Age (month)	Weight (g)	Stent retention time (days)	Operation time (min)
1	M	2.6	5,680	29	3
2	M	3	5,400	30	4
3	M	3.2	5,120	35	3
4	M	3	5,300	32	5
5	M	3.2	5,420	30	3
6	M	2.6	4,500	28	2
7	M	2.8	5,680	30	2
8	M	4	6,400	35	4
9	M	3.8	6,480	33	3
10	M	3	6,040	32	3
11	M	6	7,100	30	5
12	M	4.5	6,400	29	3
13	M	3.4	5,540	30	4
14	M	5.7	6,850	32	2
15	M	6	7,350	33	6

## Discussion

Ureteral stents have been widely used in upper urinary tract surgery since the first report by Hepperlen et al. in 1978 (4). The DJUS is the most commonly used stent, and it is mainly used in surgeries for managing congenital ureteral malformations in children, such as ureteropelvic junction obstruction, ureterovesical junction obstruction and vesicoureteral reflux. When the hydroneurosis grade reached Ⅲ or above, and gradual thinning of the renal cortex was detected by serial ultrasound, especially a finding of <40% was obtained on diuretic renal scintigraphy, even infants needed active surgical treatment to prevent irreversible damage to renal function. Percutaneous nephrostomy and insertion of a DJUS into the renal pelvis through retrograde cystoscopy had been reported in the literature. However, there were some disadvantages such as secondary operation, easy infection, and recurrence after DJUS removal.

Pyeloplasty has become the gold standard in the treatment of ureteropelvic junction obstruction, since it was first described by Anderson and Hynes in 1949. With innovations in surgical instruments and suture materials, open, laparoscopic, and robot-assisted pyeloplasties have become more popular and safe for infants (5, 6). Hydronephrosis was significantly relieved after the operation, and maximum renal function was retained. Chandrasekharam et al. (7) showed that the improvement in renal function after laparoscopic pyeloplasty was better among infants than in older children. Regardless of the surgical method chosen, the DJUS is placed in the ureter to avoid anastomotic stricture, decrease postoperative leakage, maintain reliable urinary drainage, and facilitate anastomosis. The DJUS was removed by a forcep under cystoscopy 4–6 weeks after the operation, which has the advantages of direct vision and a 100% success ratec. However, some patients, especially male infants, were still too young and their urethra was too small for cystoscopy with the operating channel. Therefore, DJUS removal was tricky in such cases, and some investigators attempted to remove DJUS without cystoscope. Kajbafzadeh et al. (8) inserted the DJUS in the feeding tube and fixed it using a proline suture during the open pyeloureteroplasty, then, they secured the feeding tube to the external skin via an additional incision. Hadley et al. (9) inserted a kidney internal splint stent catheter into the ureter and one end was left on the back of the patient through a renal puncture during laparoscopic pyeloureteroplasty. Both of the aforementioned articles indicated that the ureteral stent could be removed in the clinic without cystoscopy by removing the exposed tube, because it was connected to the ureteral stent. However, there were some disadvantages of this approach, such as additional trauma, unintended stent removal, inconvenient nursing, and urinary tract infection would be increased due to the stents wad exposed outside the body. Lin et al. (2) and Shao et al. (3) used a simple self-made device comprising a monofilament suture was tied to the end of a 5Fr feeding tube to form an open loop, and removal the stent by noncystoscopic just received minimal nitrous oxide inhalational anesthesia or administered orally chloral hydrate of 0.5 ml/kg. However, two to three attempts were required for stent removal in approximately 13% of the patients treated thusly, and failure in some cases eventually necessitated cystoscopy or other methods. Further, attention needs to be paid to the potential risks associated with this approach, in that the feeding tube may become tangled to form a knot when it is in the bladder and multiple attempts might be required to untangle it, which in turn might injure the urethra, especially in infants with a small urethra. Kajbafzadeh et al. (1) used an extraction string, which was fixed on the DJUS, sutured to the subcoronal skin in male children or the inner surface of the labia majora in girls through the urethra. The stent was removed by pulling the string. However, the DJUS slipped unexpectedly in some cases, moving the suture inadvertently causes pain, and the suture in the urethra causes discomfort. The use of magnetic DJUS in children has also been reported (10). However, it is difficult to push the magnet through the ureterovesical junction, and the approach is only suitable for retrograde catheterization cases, such as vesico-ureteral reimplantation, which limits its application. Furthermore, magnetic DJUSs are more expensive (11).

Consequently, safe and easy removal of DJUS among patients in whom cystoscopy can be used without a forceps channel is an urgent problem due to some cystoscopes without matching sheaths. We drew inspiration from the process of roping horses with horse poles. Thus, we folded a 4-0 prolene suture to form a coil, which was achieved easily because of suture hardness. Then the coil was placed into the cystoscope sheath to make a device similar to the horse pole. Due to the smooth nature of the prolene suture, cystoscopy was not affected, and the prolene suture could be tightened freely. We immediately made a training device with a 3-mm trocar, a balloon filled with water, and a DJUS to simulate the urethra and bladder. After several training sessions, the DJUS could be removed smoothly with this approach. Therefore, we communicated this idea and related risks to the patients' parents. The parents agreed to go through with the procedure and provided signed informed consent. Then, we used the technique to remove the 15 DJUS (average procedure time: 3.5 ± 1.2 min) in 15 children, whose average age was 3.78 ± 1.2 months, without causing bladder or urethra injury.

Although we used additional cystoscopy to remove the DJUS compare with the other methods reported in the literature, the whole operation process was performed using the sheath and under cystoscopy. We believe that with this technique, there will be no damage to the bladder and urethra if the cystoscope is used correctly. Since infant tissue is too delicate to allow repeated procedures, minimizing blind operation is the safest approach. Our technology precisely follows such principles.

In summary, the key point of the new technique is to push the folded prolene coil into the bladder approximately 1 cm after the end of the DJUS. If there is a large amount of floc in the bladder, which leads to unclear visualization, it is necessary to rinse the bladder with normal saline until it can be clearly visualized. When the coil traps the end of the DJUS, it is not urgent to tighten the prolene coil. It is necessary to adjust the cystoscope angle along the radian of the stent, so that the coil can cover the stent for at least 1 cm. Then, the prolene suture is tightened; meanwhile, the cystoscope is pushed forward to prevent slippage between the prolene coil and DJUS, until the coil fixes the stent into the sheath. To accurately and rapidly grasp the DJUS, complete training in the laboratory in advance is a must. Fortunately, physicians with cystoscope operation experience can master the technique with two to three training sessions.

## Conclusion

We described a safe, simple, and reliable procedure for removing ureteral stents, DJUSs, in small infants by using prolene suture-assisted cystoscopy. This technique is also suitable for patients in whom the grasping forceps cannot pass through the matching cystoscope sheath.

## Data Availability

The raw data supporting the conclusions of this article will be made available by the authors, without undue reservation.
